# Supplementary material for: Causal Relationship between Aging and Anorexia Nervosa: A White-Matter-Microstructure-Mediated Mendelian Randomization Analysis
Source: Biomedicines. 2024 Aug 16;12(8):1874. doi: 10.3390/biomedicines12081874 (PMC11351342; doi:10.3390/biomedicines12081874)
Supplement: Supplementary file 1 [file biomedicines-12-01874-s001.zip › Supplementary Figure/Figure S4/Singe SNP plot of MR analysis for TL to ubm-b-2014.pdf]

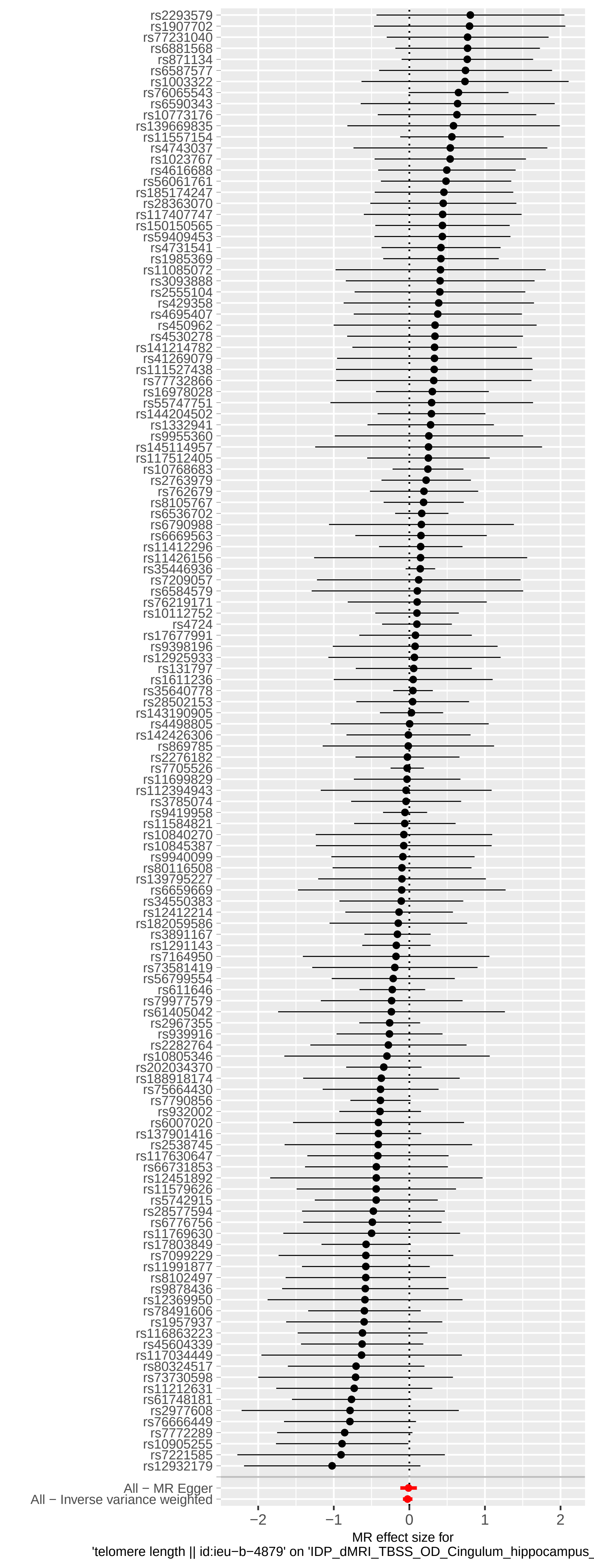
rs2293579
rs1907702
rs77231040
rs6881568
rs871134
rs6587577
rs1003322
rs76065543
rs6590343
rs10773176
rs139669835
rs11557154
rs4743037
rs1023767
rs4616688
rs56061761
rs185174247
rs28363070
rs117407747
rs150150565
rs59409453
rs4731541
rs1985369
rs11085072
rs3093888
rs2555104
rs429358
rs4695407
rs450962
rs4530278
rs141214782
rs41269079
rs111527438
rs77732866
rs16978028
rs55747751
rs144204502
rs1332941
rs9955360
rs145114957
rs117512405
rs10768683
rs2763979
rs762679
rs8105767
rs6536702
rs6790988
rs6669563
rs11412296
rs11426156
rs35446936
rs7209057
rs6584579
rs76219171
rs10112752
rs4724
rs17677991
rs9398196
rs12925933
rs131797
rs1611236
rs35640778
rs28502153
rs143190905
rs4498805
rs142426306
rs869785
rs2276182
rs7705526
rs11699829
rs112394943
rs3785074
rs9419958
rs11584821
rs10840270
rs10845387
rs9940099
rs80116508
rs139795227
rs6659669
rs34550383
rs12412214
rs182059586
rs3891167
rs1291143
rs7164950
rs73581419
rs56799554
rs611646
rs79977579
rs61405042
rs2967355
rs939916
rs2282764
rs10805346
rs202034370
rs188918174
rs75664430
rs7790856
rs932002
rs6007020
rs137901416
rs2538745
rs117630647
rs66731853
rs12451892
rs11579626
rs5742915
rs28577594
rs6776756
rs11769630
rs17803849
rs7099229
rs11991877
rs8102497
rs9878436
rs12369950
rs78491606
rs1957937
rs116863223
rs45604339
rs117034449
rs80324517
rs73730598
rs11212631
rs61748181
rs2977608
rs76666449
rs7772289
rs10905255
rs7221585
rs12932179
All − MR Egger
All − Inverse variance weighted
−2
−1
0
1
2
MR effect size for
'telomere length || id:ieu−b−4879' on 'IDP_dMRI_TBSS_OD_Cingulum_hippocampus_
